# Effect of one year of a gluten-free diet on the clinical evolution of irritable bowel syndrome plus fibromyalgia in patients with associated lymphocytic enteritis: a case-control study

**DOI:** 10.1186/s13075-014-0421-4

**Published:** 2014-08-27

**Authors:** Luis Rodrigo, Ignacio Blanco, Julio Bobes, Frederick J de Serres

**Affiliations:** Gastroenterology, Central University Hospital of Asturias (HUCA), Celestino Villamil, s/n, 33006 Oviedo, Asturias Spain; Biomedical Research Office of the Principality of Asturias, FICYT, C/ Rosal 7 bis, 33009 Oviedo, Asturias Spain; Medicine Department, Psychiatry Area, University of Oviedo, Juan Claveria 6, 33006 Oviedo, CIBERSAM, Asturias Spain; National Institute of Environmental Health Sciences, Research Triangle Park, Durham, NC 27709-2233 USA

## Abstract

**Introduction:**

Irritable bowel syndrome (IBS), lymphocytic enteritis (LE) and fibromyalgia syndrome (FMS) are three common disorders. Since a gluten-free diet (GFD) has been shown to be helpful in LE, we aimed to assess its effect in a series of LE patients also diagnosed with IBS and FMS.

**Methods:**

The study sample comprised 97 IBS plus FMS adult females, of whom 58 had LE (Marsh stage 1), and 39 had a normal duodenal biopsy (Marsh stage 0). All patients fulfilled the Rome III and American College of Rheumatology 1990 criteria. All participants followed a GFD, the effectiveness of which was assessed by changes in the results of several tests, including those of the Fibromyalgia Impact Questionnaire (FIQ), the Health Assessment Questionnaire (HAQ), tender points (TPs), the Short Form Health Survey (SF-36), and the Visual Analogue Scales (VAS) for gastrointestinal complaints, pain and fatigue.

**Results:**

At baseline, all patients had a poor quality of life (QoL) and high VAS scores. After one year on a GFD, all outcome measures were somewhat better in the Marsh stage 1 group, with a mean decrease of 26 to 29% in the TPs, FIQ, HAQ and VAS scales, accompanied by an increase of 27% in the SF-36 physical and mental component scores. However, in the IBS plus FMS/Marsh stage 0 group, the GFD had almost no effect.

**Conclusions:**

This pilot study shows that a GFD in the LE-related IBS/FMS subgroup of patients can produce a slight but significant improvement in all symptoms. Our findings suggest that further studies of this subject are warranted.

## Introduction

Irritable bowel syndrome (IBS), fibromyalgia syndrome (FMS) and lymphocytic enteritis (LE) are three common disorders that can occur in the same subject. Specifically, FMS occurs in 20 to 32% of people with IBS [1]. IBS has been found in 32 to 70% of people with FMS [2,3], and LE has been found in 16% of people with IBS, and in 56% of people with both IBS and FMS [4,5].

IBS is a functional gastrointestinal disorder, characterised by the presence of chronic abdominal pain or discomfort, and changes in the intestinal habit, bloating and abdominal distension. FMS is a chronic syndrome characterised by widespread pain, generalised tender points, fatigue, restless sleep, and various other symptoms suggestive of central nervous system dysregulation. Both diseases are prevalent chronic central sensitisation disorders respectively classified according to the Rome III [6] and the 1990 American College of Rheumatology (ACR) criteria [7], once other typified diseases have been ruled out by appropriate studies.

On the other hand, LE (also known as lymphocytic duodenosis, or Marsh stage 1, when it is associated with coeliac disease (CD)) is a common pathological finding in duodenal biopsies, especially of adults, and is characterised by the increased infiltration of intraepithelial lymphocytes (IELs) above 25% of the normal values, based on counts of 100 epithelial cells, and normal villous architecture [8]. It is becoming increasingly accepted that LE belongs within the broad spectrum of histological abnormalities observed in CD [9]. In fact, patients with CD have varying degrees of damage to their small intestinal mucosa, ranging from LE with normal villous structure to severe villous atrophy (Marsh stage 3). In addition, more IELs may be the earliest pathological change to occur, following gluten challenge, and may even be the only sign of gluten sensitivity [10]. Although LE has been considered by some to be a latent CD, its aetiology cannot be clarified in about one-third of cases. Indeed, a prospective study of the aetiology of LE in 100 patients estimated that at least 16% of patients with this condition may actually have CD [11]. However, a more recent Spanish prospective study of 90 consecutive patients with LE and clinical symptoms of CD reached a final diagnosis of gluten-sensitive enteropathy (GSE), alone or associated with *Helicobacter pylori* (HP) infection, in 43% of patients [12]. In addition to gluten sensitivity, LE may be related to non-steroidal anti-inflammatory drug (NSAID) intake, intolerance to non-gluten food protein (for example, cow’s milk, eggs, peanuts, soya), autoimmune disorders (for example, thyroiditis, type I diabetes, rheumatoid arthritis, psoriasis, multiple sclerosis, systemic lupus erythematosus), inflammatory and infectious intestinal tract disorders (for example, Crohn’s disease, bacterial overgrowth, HP, tropical sprue, and *Giardia lamblia*, *Cryptosporidium* and viral infections), and T-cell intestinal lymphoma [13,14]. Two or more aetiological factors are often associated, the most frequent of these being NSAIDs, CD and HP infection [11,12].

Some researchers have reported that patients with LE (that is, Marsh stage 1) may have the same type of gastrointestinal and extra-intestinal symptoms as patients with CD-related villous atrophy [15], and notably good responses to a gluten-free diet (GFD) have also been observed in Marsh Stage 1 patients [16–18].

Accordingly, since GSE may be responsible for LE in 16 to 43% of cases and, furthermore, Marsh stage 1 patients may benefit from a GFD, especially if they have extraintestinal manifestations [19], the aim of this study was to analyse the changes in the scores of several Health-Related Quality of Life (HR-QoL) questionnaires and the Visual Analogue Scales (VAS), before and after (pre-post) treatment with a GFD for one year, in two groups of patients recruited by case finding for CD [4]. One group consisted of subjects with concurrent IBS, FMS and LE (Marsh stage 1), and the other of subjects with IBS and FMS with normal intestinal mucosa (Marsh stage 0).

## Methods

As has been reported in two recent articles consecutively published by our group [4,5], during the six-year period between 2007 and 2012, we prospectively studied by CD screening detection a total of 442 consecutive Caucasian patients who had been referred to the Gastroenterology Outpatient Clinic of the Central University Hospital of Asturias, HUCA (Oviedo, Spain) for a variety of chronic gastrointestinal and systemic symptoms.

Details of subject selection with inclusion and exclusion criteria, analytical tests performed, HR-QoL questionnaires used, and technical details of collection, staining and interpretation of gastrointestinal biopsies were extensively described in the aforementioned papers [4,5].

Briefly, 229 out of 442 subjects volunteered to take part in this study and gave their specific signed informed consent. Rome III criteria for IBS diagnosis [6] and the ACR 1990 criteria for FMS classification [7] were applied to each patient on their first visit. A comprehensive medical history was taken, patients were given a thorough physical examination, and complete laboratory haematological and biochemical screening was carried out. The study was approved by the Research and Ethics Committee of the HUCA, following the principles of the modified Declaration of Helsinki.

Cell blood count (CBC) and coagulation studies were performed with an automated Abbott Hematology Analyzer (Cell Dyn 3500, Abbott Diagnostics, Santa Clara, CA, USA) and the Coagulation Analyzer ACL 3000 (Menarini Diagnostics, Florence, Italy), respectively. Biochemical tests were performed with a Hitachi Modular automated analyzer SXA-PPBD (Roche, Basel, Switzerland) using enzymatic or kinetic methods to measure blood urea, glucose, total protein, albumin, C-reactive protein (CRP), calcium, folate, vitamin B-12, creatinine, creatine kinase (CK), rheumatoid factor (RF), lipid profile, liver function, immunoglobulin levels (IgG, IgA and IgM), iron metabolism, thyroid function, and urine with microscopic examination of the sediment. Anti-nuclear antibodies (ANAs) and anti-thyroid peroxidase (anti-TPO) antibodies were measured in each participant. In cases with altered liver function tests (LFTs), anti-mitochondrial antibodies (AMAs) were assessed by indirect immunofluorescence assay in the Hep-20-10 cell line (Euroimmun, Lübeck, Germany). Anti-IgA tissue transglutaminase subtype 2 (tTG) antibodies were measured with an ELISA kit from Phadia Diagnostics (Uppsala, Sweden). Major histocompatibility complex class II (HLA-DQ2) genetic markers (DQA1*0501 and DQB1*0201 alleles) were determined by a polymerase chain reaction (PCR) with a ProtransR HLA Celiac Disease Domino System (Protrans, Ketsch, Germany) kit. The HLA-DQ8 haplotype was identified in a single negative HLA-DQ2 patient with duodenal villous atrophy.

An upper gastrointestinal endoscopy with at least four duodenal biopsies was performed following the usual methods in all patients included in the case finding/screening. Samples were routinely stained with haematoxylin and eosin (H&E) and with anti-CD3 immunohistochemical monoclonal antibodies to verify the presence and count the number of IELs. These were in turn quantified in relation to 100 epithelial cells. Samples were studied by two expert pathologists from the HUCA, and classified into the following types: Stage 0, normal duodenum; Stage 1, increased IEL infiltration with a total count of ≥25%; Stage 2, crypt hyperplasia and presence of diffuse chronic inflammatory infiltration of the lamina propria; Stage 3, villous atrophy, subdivided into three categories: (a) mild, (b) moderate and (c) severe, according to the histological classification of CD described by Marsh in 1992 [20] and subsequently modified by Oberhüber *et al*. [21].

HP from endoscopic gastric biopsies was systematically investigated. Antibiotics, proton pump inhibitors (PPIs), drugs containing bismuth and H2 blockers were withdrawn during the two preceding weeks. Two antral biopsies were immediately taken for a rapid urease test (Pronto Dry Kit, Pentland Medical Ltd, Edinburgh, UK). The remaining samples were used for histopathological examination (that is, routine H&E staining, Giemsa staining and immunohistochemistry using polyclonal anti-HP antibody) and microbial culture. Positive cases received triple therapy involving a 14-day treatment with PPI (40 mg twice a day (bid)), clarithromycin (500 mg bid), and amoxicillin (1,000 mg bid). After completing treatment, HP eradication was confirmed four to six weeks later with a rapid urease breath test [22,23].

In non-responders to usual therapies, doubtful cases or those suspected of having other associated organic illnesses, a specific breath test was performed to rule out possible lactose intolerance or a small bowel bacterial overgrowth. Faecal cultures were grown, when required, to exclude the possibility of parasitic infections in some patients. A total colonoscopy was carried out and random colonic biopsies taken from patients with persistent diarrhoea to rule out the possible presence of microscopic colitis.

An immunological faecal occult blood test (iFOBT) was done in patients aged over 50 years and in those with a positive family history of colon cancer in first-degree relatives. If the iFOBT was positive the study was completed with a total colonoscopy.

Tender points (TPs) were identified and their frequency determined by digital pressure of the 18 anatomical locations recommended by the ACR 1990 study [7].

To measure physical, mental, psychological, and social functioning, each patient completed the Spanish versions of the self-administered Fibromyalgia Impact Questionnaire (FIQ), Health Assessment Questionnaire (HAQ) and Short Form Health Survey (SF-36) [24–26].

The FIQ comprises a 10-item scale that evaluates the global impact of the illness on the patient, with a total score ranging between 0 and 80 points, derived from various questions about physical functioning, work status level, degrees of depression and anxiety, sleep alterations, severity of pain, stiffness, fatigue and the perception of wellbeing. Total FIQ scores were divided into three categories, ranging from 0 to 39 (mild), 40 to 59 (moderate), and 60 and over (severe) [24].

The HAQ test is a 20-item disability scale of various 4-point ordinal measures of a patient’s difficulty and need for help and assistive devices in several daily activities, with higher scores indicating greater impairment (0 = able to do without any difficulty, 1 = some difficulty, 2 = much difficulty, and 3 = unable to do) [25].

The 36 items of the short form SF-36 questionnaire cover eight health domains: (1) physical functioning (PF); (2) role limitations by physical problems (RP); (3) bodily pain (BP); (4) general health perceptions (GH); (5) vitality (energy/fatigue) (VT); (6) social functioning (SF); (7) role limitations due to emotional problems (RE); (8) mental health/emotional well-being perception (MH). These eight measures are used to calculate two weighted aggregate scores: the physical and mental component summaries (PCS and MCS). Scores may take values between 0 and 100, higher ones indicating better health. No threshold values of SF-36 scores have so far been established that classify the impact of a disease. For reference, the published values for adults in the general Spanish population are 73 ± 28 for the PCS and 74 ± 24 for the MCS [26].

To evaluate the severity of digestive symptoms and the amount of pain and fatigue experienced by patients, three types of VAS were used [27–29].

Participants were then assigned to one of two groups: the first consisted of 104 subjects who fulfilled the Rome III criteria for IBS diagnosis and the ACR 1990 criteria for FMS classification constituted the IBS plus FMS group, and the second one of 125 unrelated and age- and sex-matched subjects, fulfilling the Rome III criteria for IBS but not those of the ACR 1990 criteria for FMS, comprised the IBS group. Seven cases with villous atrophy (7%), 58 with LE (56%) and 39 with a normal intestinal biopsy (37%) were found in the IBS and FMS group, while in the group of IBS without FMS we found 2 subjects with villous atrophy (2%), 20 with LE (16%) and 105 with a normal biopsy (84%). All patients with intestinal atrophy were successfully treated with DSG [5].

The current study was finally limited to 58 cases with IBS, FMS and LE (Marsh stage 1), and to 39 cases from the same group with IBS and FMS who had a normal intestinal biopsy (Marsh stage 0). Patients in both groups were treated with a GFD for one year. The diet was followed as strictly as possible, in order to try to keep daily gluten consumption to a minimum.

### Statistical analysis

Continuous parameters were summarised as means and standard deviations, and medians and ranges. Qualitative variables were summarised as percentages. Kruskal-Wallis analysis of variance (ANOVA) and contingency tests were then undertaken. Normally distributed continuous variables were analysed with Student’s *t* test for unpaired samples, and ANOVA followed by a *post hoc* Fisher’s test. All statistical tests were two-sided, with significance concluded for values of *P* <0.05. Statistical calculations were performed with SPSS 15.0 (SPSS Inc., Chicago, IL, USA).

## Results

### Description of the cohorts

Baseline demographic, gender, social, clinical, serological and genetic characteristics of the IBS/FMS Marsh stage 0 (normal mucosa) and stage 1 (LE) cohorts are shown in Table 1. Eighty-nine per cent of patients were women. The mean age of the complete sample was 50 ± 8 years. There were no significant differences in these numbers between the cohorts. Patients in both cohorts had a slightly high body mass index (BMI), indicative of being overweight, especially among the constituents of the Marsh stage 1 group. No differences in the level of education or in employment status were found.

**Table 1:** **Baseline characteristics of the cohorts**

	**All subjects n = 97**	**Normal mucosa (Marsh stage 0) n = 39**	**Lymphocytic enteritis (Marsh stage 1) n = 58**	***P***
**Demographic characteristics**				
*Females, n (%)*	86 (89)	34 (87)	52 (90)	0.959
*Age in years, mean (SD)*	50 (8)	49 (7)	51 (9)	0.222
*BMI, kg/m* ^*2*^ *, mean (SD)*	26 (4)	25 (3)	28 (5)	<0.001
**Education level**				
*None, n (%)*	2 (2)	0	2 (3)	0.658
*Primary education, n (%)*	71 (73)	29 (74)	42 (72)	0.983
*Secondary education, n (%)*	22 (23)	10 (26)	12 (21)	0.746
*Higher education, n (%)*	3 (3)	0	2 (3)	0.658
**Employment status**				
*Employed, n (%)*	45 (46)	21 (54)	24 (41)	0.317
*Unemployed, n (%)*	20 (21)	7 (18)	13 (22)	0.782
*Homemaker, n (%)*	17 (17)	8 (20)	9 (15)	0.717
*Retired/pensioner, n (%)*	15 (15)	3 (8)	12 (21)	0.147
**First-degree relatives with CD/FMS**				
*Subjects with CD relatives, n (%)*	10 (10)	0	10 (17)	0.016
*Subjects with FMS relatives, n (%)*	4 (4)	0	4 (7)	0.248
**Gastroduodenal histopathological details**				
*IELs, mean (SD)*	18 (3)	14 (2)	35 (5)	<0.001
*Helicobacter pylori (+), n (%)*	41 (42)	17 (43)	24 (41)	0.426
**HLA haplotype**				
*HLA-DQ2 A1/B1, n (%)*	46 (47)	15 (38)	31 (53)	0.214
*HLA-DQ2 (−), n (%)*	15 (15)	4 (10)	11 (19)	0.381
**Serum autoantibodies**				
*Anti tTG-2, U/ml, mean (SD)*	0.7 (0.6)	0.5 (0.1)	0.9 (0.7)	<0.001
*RF, IU/m, mean (SD)*	12.8 (1.7)	12.6 (0.9)	12.9 (2.1)	0.552
*Anti-TPO (+), U/ml, mean (SD)*	30 (111)	20 (30)	51 (140)	0.115
*ANAs (+), n (%)*	26 (27)	3 (8)	23 (40)	0.001
*AMAS (+), n (%)*	4 (4)	0	4 (7)	0.248

Data are expressed as frequency (*n*) and percentage of the total value (%), or mean and standard deviation (SD). BMI, body mass index; IELs, intraepithelial lymphocyte count from 100 epithelial cells; anti-tTG-2, anti-tissue transglutaminase-2 antibodies; RF, rheumatoid factor; HLA-DQ2, major histocompatibility complex class II; anti-TPO, anti-thyroid peroxidase antibodies; ANAs, anti-nuclear antibodies; AMAs, anti-mitochondrial antibodies.

Ten subjects in the Marsh stage 1 group, but none in the Marsh stage 0 group, had several coeliac relatives. Four subjects in the Marsh stage 1 group with no CD family history, but none in the Marsh stage 0 group, had relatives with FMS. Approximately equal proportions (more than 40%) of subjects from the two groups were positive for HP. No differences between the cohorts were found with respect to the HLA-DQ2 haplotypes, blood titres of RF, anti-TPO, and AMAs. However, serum levels of anti-tGT-2 and ANAs were significantly higher among Marsh stage 1 patients.

### Symptoms, associated disorders and prescribed drugs

All patients complained of a combination of the following digestive symptoms: heartburn, bloating, diffuse abdominal pain/discomfort, bowel habit alteration, and constipation and diarrhoea, separately or alternating. The symptoms first appeared in the patients’ 20s, in the vast majority of cases. All complained of a number of common FMS symptoms, including widespread soft-tissue pain, abnormal fatigue, sleep disturbances, foggy mind, and so on, generally starting around two to three decades later than the onset of the gastrointestinal symptoms. However, no differences between the two groups were found with respect to gastrointestinal and systemic symptom type or duration, or in the prevalence of associated diseases.

Patients in both groups took a large number of drugs of different types. There were no differences between the groups with respect to drug prescriptions, except that some Marsh stage 1 patients received opioid patches while Marsh stage 0 patients did not (Table 2).

**Table 2:** **Symptoms, associated disorders and prescribed drugs**

**Symptoms** ***, n (%)***	**All cases n = 97**	**Normal mucosa (Marsh stage 0) n = 39**	**Lymphocytic enteritis (Marsh stage 1) n = 58**	***P***
*Widespread soft-tissue pain*	97 (100)	39 (100)	58 (100)	1.000
*Digestive complaints* ^*†*^	97 (100)	39 (100)	58 (100)	1.000
*Fatigue*	88 (91)	35 (90)	53 (91)	0.932
*Sleep disturbances*	81 (83)	29 (74)	52 (89)	0.087
*Anxiety/depression*	64 (66)	22 (56)	42 (72)	0.158
*Skin problems* ^*‡*^	48 (49)	20 (51)	28 (48)	0.934
*Foggy mind*	34 (35)	14 (36)	20 (34)	0.941
*Urinary urgency*	28 (29)	10 (26)	18 (31)	0.729
*Balance problems/dizziness*	27 (28)	10 (26)	17 (29)	0.869
*Chronic headaches* ^*⊥*^	26 (27)	5 (13)	21 (36)	0.021
*Joint stiffness*	25 (26)	9 (23)	16 (28)	0.794
*Paraesthesias*	23 (24)	9 (23)	14 (24)	0.902
**Associated disorders** *, n (%)*				
*Osteoporosis*	12 (12)	2 (5)	10 (17)	0.144
*TMJ disorder*	11 (11)	5 (13)	6 (10)	0.959
*Restless legs*	11 (11)	3 (8)	8 (14)	0.547
**Symptom duration**, y*ears, mean (SD)*				
*Gastrointestinal symptoms*	29 (6)	29 (5)	29 (8)	0.029
*Extraintestinal symptoms*	9 (2)	9 (2)	9 (2)	0.501
**Prescribed drugs** *, n (%)*				
*Analgesics* ^*^	79 (81)	34 (87)	45 (78)	0.344
*Omeprazole*	76 (78)	29 (74)	47 (81)	0.595
*Antidepressants* ^*⊥*^	65 (67)	25 (64)	40 (69)	0.780
*Pregabalin*	55 (57)	19 (49)	36 (62)	0.275
*Benzodiazepines/hypnotics*	55 (57)	20 (51)	35 (60)	0.500
*Antispasmodics/Antidiarrhoeals* ^*†*^	37 (38)	17 (38)	33 (57)	0.281
*Laxatives*	13 (13)	10 (26)	27 (47)	0.062

^†^Digestive complaints consisted of a combination of at least two of the following symptoms: bloating, heartburn, epigastric pain, diffuse abdominal pain, constipation and diarrhoea (separately or alternating); ^‡^skin problems included: itchy/dry/burning skin, dermographism, chronic urticaria, and one case of herpetiformis dermatitis; ^⊥^chronic headaches encompassed: migraines, muscle tension and combination headaches; ^*^analgesics were usually used on an irregular basis (on demand), and generally consisted of non-steroidal anti-inflammatory drugs, paracetamol, tramadol, metamizol and codeine; ^⊥^antidepressants: tricyclic antidepressants, selective serotonin reuptake inhibitors, other antidepressants; ^†^antispasmodics: mebeverine and/or otilonium bromide; ^†^antidiarrhoeals: loperamide, diphenoxylate/atropine. TMJ: temporomandibular joint.

### Comparison of global pre and post outcome measures

**Figure 1:**
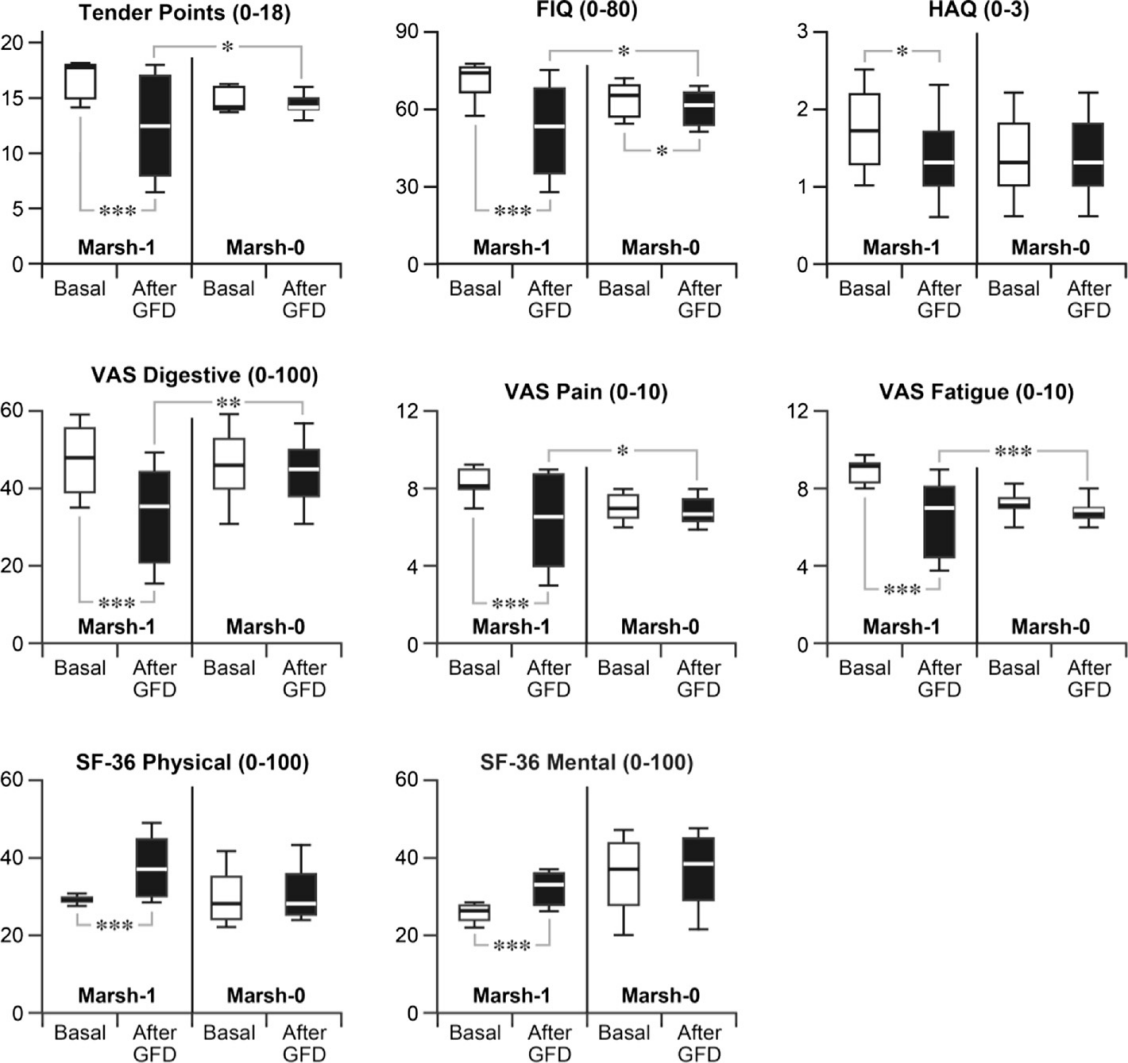
**Comparative results of clinical evaluation tests in Marsh stage 1 vs** ***.*** **stage 0 patients, at baseline and after one year of GFD.** FIQ, Fibromyalgia Impact Questionnaire; GFD, gluten-free diet; HAQ, Health Assessment Questionnaire; Marsh-1, lymphocytic enteritis; Marsh-0, normal mucosa; SF-36, Short Form Health Survey; VAS, Visual Analogue Scale. In the box-and-whisker plots, the box is delimited by the 25th and 75th percentiles, the horizontal line within the box marks the 50th percentile (median), and the whiskers indicate the 10th and 90th percentiles. ^***^ *P* <0.0001; ^**^ *P* < 0.001; ^*^ *P* <0.05.

The baseline (‘pre’) scores and those after one year of GFD (‘post’) of both groups for the TPs, FIQ, HAQ, VAS and SF-36 questionnaires are illustrated as box-and-whisker plots, which show the mean (50th percentile) and range (10th, 25th, 75th and 90th percentiles) of each outcome (Figure 1).

A very significant decrease in the mean score of TPs was observed in the Marsh stage 1 group (pre = 16.9 vs*.* post = 12.2, *P* <0.0001). In contrast, no significant changes were found in the Marsh stage 0 group (pre = 14.8 vs*.* post = 14.3).

A very significant 20-point drop in the FIQ mean score was observed in the Marsh stage 1 group (pre = 70.1 vs*.* post = 50.1, *P* <0.0001). There was also a slight drop in the FIQ score in the Marsh stage 0 group (pre = 63.4 vs. post = 61.1, *P* <0.05).

A modest but significant decrease in the HAQ score was observed in the Marsh stage 1 group (pre = 1.6 vs*.* post = 1.1, *P* <0.05), but not in the Marsh stage 0 group (pre = 1.4 vs. post = 1.4).

A highly significant decrease in the VAS digestive symptom score was observed in the Marsh stage 1 group (pre = 47.5 vs*.* post = 33.2, *P* <0.0001), whereas the change in the Marsh stage 0 group (pre = 45.9 vs*.* post = 44.0) was not significant.

There was a significant decrease in the VAS pain score in the Marsh stage 1 group (pre = 8.6 vs. post = 6.2, *P* <0.0001), but not in the Marsh stage 0 group (pre = 7.0 vs*.* post = 6.8).

There was a significant decrease in the VAS fatigue score in the Marsh stage 1 group (pre = 8.6 vs*.* post = 6.4, *P* <0.0001), but no significant change in the Marsh stage 0 group (pre = 7.1 vs*.* post = 6.8).

There was a significant increase in the SF-36 PCS in the Marsh stage 1 group (pre = 28.6 vs. post = 36.5, *P* <0.0001), but not in the Marsh stage 0 group (pre = 30.0 vs*.* post = 31.0).

A very significant increase in the SF-36 MCS was observed in the Marsh stage 1 group (pre = 25.5 vs*.* post = 32.1, *P* <0.0001), whereas there was no significant change in the Marsh stage 0 group (pre = 35.5 vs. post = 36.5).

## Discussion

The most remarkable finding in the present study was the partial but statistically significant improvement in all measured parameters after one year of continuous treatment with a GFD in patients of the IBS/FMS/LE (that is, Marsh stage 1 in terms of CD) group, compared with the modest or negligible improvements found in the group of patients with IBS/FMS and normal intestinal mucosa (that is, Marsh stage 0 in terms of CD).

Overall, after one year of sustained GFD a significant improvement (26 to 30%) in all outcome measures was achieved in the IBS/FMS/LE (Marsh stage 1) group compared with 3 to 4% in the IBS/FMS (Marsh stage 0) group. Specifically, the percentage differences in each parameter between Marsh stage 1 and stage 0 patients were: TPs, 28.0 vs*.* 3.2%; FIQ, 27.7 vs*.* 3.6%; HAQ, 28.4 vs*.* 3.4%; VAS digestive, 29.7 vs*.* 4.1%; VAS pain, 27.4 vs*.* 3.5%; VAS fatigue, 26.2 vs*.* 3.4%; SF-36 PCS, 27.3 vs*.* 3.8%; and SF-36 MCS, 27.2 vs*.* 3.7%. The improvements in the physical and mental components of the SF-36 were also significant, even though they were only 50% and 43%, respectively, of the estimated general Spanish population mean [26].

The small improvement in the scores of the IBS/FMS/Marsh stage 0 group did not have any clinical consequences. However, the larger improvement in scores obtained in the Marsh stage 1 group, though not completely resolving the symptoms of any case, did produce a reduction in the severity of the ailments, and ultimately a substantially favourable effect on patients’ HR-QoL. For example, the ostensible drop in the mean FIQ score involved a partial but appreciable improvement in the severity of FMS, from ‘severe’ to ‘moderate’. However, the apparently modest drop in the HAQ score meant that although patients continued to have difficulties performing normal daily activities, doing so involved less drudgery. The VAS for digestive symptoms changed from ‘severe’ to ‘moderate’, the VAS for pain changed from ‘very poor’ to ‘moderately tolerable’, and a similar result was obtained for the intensity of patients’ perceived exertion.

Recent findings have demonstrated that the spectrum of gluten-related disorders includes, along with CD, a new syndrome, defined as non-coeliac gluten sensitivity (NCGS) by the two Consensus Conferences held in London (2011) [30] and Munich (2012) [31]. The patients described herein share the typical clinical picture of this new syndrome, which is characterised by IBS-like gastrointestinal symptoms and a range of extraintestinal signs that also include joint/muscle pain resembling FMS. The majority of the other symptoms, reported in Table 2, are consistent with the clinical characteristics of NCGS: digestive complaints, fatigue, sleep disturbances, anxiety, depression, skin problems, cognitive dysfunction (‘foggy mind’), headache and paraesthesias. As is generally acknowledged, the diagnosis of NCGS implies the exclusion of a diagnosis of CD on the basis of negative coeliac serology (tTG and EmA) and the absence of villous atrophy in the intestinal biopsy. The histology of NCGS is characterised by normal mucosa (Marsh 0) in about 60% of cases and by LE (Marsh 1) in the remaining 40%. This concept is very important and we want to clearly underline that an isolated LE is never an expression of potential CD.

It has been shown that first-generation antigliadin antibodies (AGAs) are the only serological markers found in a significant proportion of patients with NCGS. Several authors have shown that these antibodies are present in a significant proportion (around 50%) of patients with NCGS [32–34]. We did not measure the AGAs in our patients because our study population comprises only adults, whose sensitivity and specificity are low (around 50% in both cases), making the routine use of AGAs of no diagnostic use in either patients with LE or cases of NCGS.

AGAs have been shown to be of use only in children under three years of age and have been largely superseded by anti-peptide antibodies of deamidated gliadin (anti-DGP), which are also of use in children, but not in adults.

CD is a multisystemic autoimmune disorder related to a permanent intolerance to gluten, a protein found in bread, pasta, cookies, pizza crust and many other foods containing wheat, barley, rye and oats. It affects 1 to 2% of the population, predominantly women, worldwide. People with the disorder are generally carriers of one of the two HLA-II genotypes, DQ2 and DQ8. In these subjects, gliadin peptides trigger an aberrant immune response, resulting in the production of tTG autoantibodies and an immune-mediated chronic inflammation of the small bowel mucosa that is associated with an increase in the number of lymphocytes in the epithelium, and facilitates the appearance of elongated hyperplastic crypts, cuboidal enterocytes with damaged brush borders, a dense infiltration of the lamina propria by lymphocytes and plasma cells, a high IEL count on the surface epithelium, and ultimately, villous atrophy featuring shortened or totally absent villi [20,21]. These histological abnormalities can develop gradually in subsequent phases, and although the onset of symptoms is usually characterised by a time lag of months or years following gluten introduction, they can appear at any age during adulthood and at any histopathological stage, with gastrointestinal and/or extraintestinal systemic manifestations. However, some coeliac subjects may remain oligosymptomatic or asymptomatic throughout their lifetime. Notably, a GFD brings about complete clinical remission and full recovery of the intestinal damage in the vast majority of CD patients [35].

The diagnosis of CD has conventionally been based on the histopathological changes of the proximal small bowel mucosa, mucosal atrophy being considered a fundamental change with which CD may be diagnosed with certainty, and on this basis, a GFD prescribed [35,36]. The strict application of this criterion would prevent the inappropriate prescription of GFD, a measure which is potentially expensive, difficult to maintain and socially restrictive [11]. However, CD has a wide spectrum of histological abnormalities, ranging from LE with normal villous architecture to flattened mucosa. Interestingly, these morphological changes occur independently of the patients’ symptoms and the interpretation of the clinical significance of LE remains controversial [37,38].

Some authors believe that since mucosal damage can develop gradually, and that some LE patients can present clinical symptoms of CD before major histological abnormalities appear, it might be unethical not to recommend a GFD to LE patients whose clinical data suggest CD [16–19]. According to this argument, the recognition of CD at this early stage would be important and very useful because these patients may develop symptoms and complications that could be improved or prevented by a GFD [39].

Identifying CD in patients with LE is a challenge for clinicians. It is best done by carrying out several specific serological and genetic diagnostic tests on a routine clinical basis to obtain a differential diagnosis of the various clinically suspicious entities, such as functional dyspepsia and IBS. This enables the identification of gluten-related histological changes, which can inform the decision to prescribe a GFD, and the assessment of the clinical/histological improvement produced by the diet [40,41].

Further evidence is required that the diagnosis of CD should always involve testing for class 2 HLA genotypes encoding DQ2 or DQ8, and tTG and⁄or anti-endomysial antibodies (EMAs) in the presence of normal IgA levels [11]. A retrospective comparative study of 124 patients with LE and 454 CD patients found that the LE cohort differed significantly in terms of the HLA type (51% of LE patients were negative for DQ2 or DQ8, whereas only 2% CD patients had neither DQ2 nor DQ8), in tGT and EMA serum antibodies (12% vs*.* 87% and 0% vs. 87%, respectively), and with respect to several clinical features (for example, LE was less frequently associated with anaemia, malaise or skin disorders, or with a positive family history of CD) [42]. However, patients with LE who are on the CD spectrum generally have negative coeliac serology (measured by tGT and EMA), and their positivity is strongly correlated with the severity of mucosal damage [43]. On the other hand, although the HLA DQ2 or DQ8 phenotypes are closely associated with CD (occurring in up to 98% of cases), they are not specific enough to establish a diagnosis of CD, since they are also found in over 25% of the general population. In any case, the absence of the genes predisposing for CD (the HLA-DQ2 and/or HLA-DQ8 haplotypes) makes CD very unlikely, because they have a high negative predictive value for this disease [35,44].

The presence of intestinal deposits specific for tTG2 IgA is a more sensitive marker of CD than serum tTGA, but is less specific, since more than 10% of controls have positive deposits. The technique for measuring it is not currently available in most clinical laboratories, and its clinical utility is not well established [45,46].

In contrast to duodenal TG2 IgA deposits, tTG antibodies in the supernatant of cultured duodenal biopsies and duodenal aspirates show greater specificity for the diagnosis of GSE, and thus, when positive, are of great value in the differential diagnosis of LE due to GSE from other forms of LE. Again, the clinical utility of this technique is not yet well established [47].

The immunophenotyping pattern of IELs in CD shows a characteristic feature of elevated CD3+ IELs (αβ and γδ TcR) and decreased CD3- IELs [48,49]. The increase in γδ- IELs is a pathognomonic (never described in other gastrointestinal disorders) and irreversible feature of CD patients, even in those who are GFD-compliant. Other cytometric abnormalities described in CD patients are the decreases in CD3^−^, CD103^+^, CD7^+^ and CD45^+^ IELs [19]. Nevertheless, this promising and sophisticated technique is not available in most clinical laboratories, so its true value has yet to be demonstrated.

A gluten challenge can cause further mucosal deterioration in patients with potential CD in whom initial small intestinal biopsies reveal only minor abnormalities. This was demonstrated by a prospective study of 38 patients with Marsh stage 1 lesions who underwent a gluten challenge (30 g per day of gluten added to a normal diet for eight weeks) [50]. This worsened the histological lesions in 12 patients (32%), while lesions remained unchanged in the other 26 patients. Additionally, the 12 patients who experienced worsening of their duodenal lesions with gluten provocation subsequently experienced symptoms of relief when placed on a GFD. In light of these results it was proposed that a gluten challenge could be useful for identifying gluten-sensitive patients. However, this strategy is time-consuming, expensive and aggressive, because it requires repeating small intestinal biopsies, so it is not recommended in clinical practice.

Finally, the diagnosis of CD-related LE could be based on a clinical and histological response to a GFD in individuals with CD-associated symptoms, positive serology (tGT and EmA), and compatible HLA coeliac predisposition genes. A drawback of this strategy is that it is time-consuming and requires good adherence to the follow-up by patients and physicians. Nevertheless, it might be a reasonable alternative in cases in which the doctor lacks sufficient means to ensure an accurate diagnosis, and in which the patient accepts testing with a GFD [51].

While there is currently no consensus among gastroenterologists about whether to treat all Marsh stage 1 patients with a GFD, there are a few published clinical trials supporting the use of the GFD in selected patients with LE [16–18]. For example, in 2003 Tursi and Brandimarte [16] assessed the benefit of a GFD in 35 young Italian patients with small bowel mucosal abnormalities (lymphocytosis type) with or without crypt hyperplasia (Marsh stages 1 to 2) and various symptoms suggestive of gluten sensitivity (for example, diarrhoea, abdominal pain, flatulence, weight loss, chronic fatigue, and so on). Remarkably, a dramatic clinical improvement in all symptoms was observed, although not always accompanied by an improvement in the histological lesions, in almost all patients after 8 to 12 months on a sustained GFD. This finding, in the opinion of the study’s authors, supports the suspicion that these patients were sensitive to gluten, and would justify starting them on a GFD. This study also found that IgG AGA, IgA EMA and IgA tGT antibodies, with 5%, 8% and 17% positivity, respectively, were poor indicators of CD in these ‘borderline’ LE cases. The reason for the failure may be the low prevalence of these antibodies in patients with mild histological lesions but without villous atrophy [43].

A subsequent prospective, randomised, controlled clinical trial in 23 EMA-positive patients with LE inflammation (Marsh stage 1/2) was performed by Kurppa *et al*. in Finland [17]. Patients were randomised either to continue on a normal gluten-containing diet or to adhere to a strict GFD. In the gluten-containing diet group the small bowel mucosal villous architecture deteriorated in all subjects, and symptoms and high serum antibody levels did not appreciably change. In contrast, the symptoms of subjects in the GFD group were alleviated, their antibody titres decreased and their mucosal inflammation diminished. When the trial was completed, all participants chose to continue on a life-long GFD. One year later, Kurppa *et al*. reported the results of another prospective trial with GFD in 73 EMA-positive adults, which showed Marsh stages 1/2 and 3 changes in duodenal biopsies. They found that patients who were Marsh stage 1/2 at baseline had more digestive symptoms and lower bone density than those with atrophy, and that the symptoms and bone mineralisation of most of them improved when they were placed on a GFD [18].

To our knowledge, there are no published therapeutic trials apart from the present one that examine the effect of a GFD in patients with concomitant LE, IBS and FMS, so comparisons with other studies are not possible. Nevertheless, we believe it is important to point out that our patients were suffering from severe FMS, a very negative intercurrent disease, because it is widely accepted that this disorder is highly refractory to all available therapies. In this regard, a classic study of patients with established FMS seen at several American rheumatological centres showed that they had markedly abnormal scores for pain, functional disability, fatigue, sleep disturbance and psychological status, and that these values did not change substantially over a prolonged follow-up period. Values at the first assessment predicted final values; half the patients were dissatisfied with their health, and most of them rated their health as fair or poor [52].

An example of the difficulty of controlling the FMS associated with CD is provided by a recent study performed in Italy, which found 13 patients with FMS (11.4%) among 114 CD subjects. Based on the subjective testimony of these patients, their widespread pain symptomatology began several years before the diagnosis of CD and their FMS symptoms were minimally improved by, or did not benefit at all, from the GFD [53].

Nevertheless, a more recent trial by our group in seven screening-detected CD adult females, also categorised as severe IBS and FMS patients, demonstrated that the TPs, FIQ, HAQ, VAS scales, and SF-36 PCS and MCS scores were significantly improved by 50 to 60%, accompanied by a decrease in tTG-2 to normal values, after one year of the GFD. These results suggest that a significant positive outcome was obtained with the GFD, although the symptoms did not completely disappear in any of the cases [5].

## Conclusions

In summary, although there is currently little scientific evidence, and we are not able to draw definite conclusions about the effectiveness of the GFD in FMS patients, it seems reasonable to infer that GFD is not appropriate for patients with a normal intestinal biopsy (Marsh stage 0), and that more accurate, randomised, double-blind, placebo-controlled studies of greater statistical power are required to assess this recommendation before applying it widely to patients with FMS and LE (Marsh stage 1).

## Abbreviations

ACR: American College of Rheumatology
AGAs: antigliadin antibodies
AMAs: anti-mitochondrial antibodies
ANAs: anti-nuclear antibodies
anti-TPO: anti-thyroid peroxidase antibodies
bid: twice a day
CD: coeliac disease
EMA: anti-endomysial antibodies
FIQ: Fibromyalgia Impact Questionnaire
FMS: fibromyalgia syndrome
GFD: gluten-free diet
GSE: gluten-sensitive enteropathy
HAQ: Health Assessment Questionnaire
HLA: human leukocyte antigens
HP: *Helicobacter pylori*
HR-QoL: Health-Related Quality of Life
IBS: irritable bowel syndrome
IELs: intraepithelial lymphocytes
iFOBT: immunological faecal occult blood test
LE: lymphocytic enteritis
MCS: mental component summary
NCGS: non-coeliac gluten sensitivity
NSAIDs: non-steroidal anti-inflammatory drugs
PCS: physical component summary
PPIs: proton pump inhibitors
RF: rheumatoid factor
SF-36: Short Form Health Survey
TPs: tender points
tTG: tissue transglutaminase
VAS: Visual Analogue Scale
